# Morphology, Molecular Identification, and Pathogenicity of Two Novel *Fusarium* Species Associated with Postharvest Fruit Rot of Cucurbits in Northern Thailand

**DOI:** 10.3390/jof8111135

**Published:** 2022-10-27

**Authors:** Surapong Khuna, Jaturong Kumla, Tanapol Thitla, Wipornpan Nuangmek, Saisamorn Lumyong, Nakarin Suwannarach

**Affiliations:** 1 Research Center of Microbial Diversity and Sustainable Utilization, Chiang Mai University, Chiang Mai 50200, Thailand; 2 Department of Biology, Faculty of Science, Chiang Mai University, Chiang Mai 50200, Thailand; 3 Faculty of Agriculture and Natural Resources, University of Phayao, Muang Phayao 56000, Thailand; 4 Academy of Science, The Royal Society of Thailand, Bangkok 10300, Thailand

**Keywords:** cucurbit, fruit rot, fungal disease, new species, pathogen identification, taxonomy

## Abstract

Fruit rot of cucurbits caused by several pathogenic fungi has become an important postharvest disease worldwide. In 2022, fruit rot on watermelon (*Citrullus lanatus*) and muskmelon (*Cucumis melo*) was observed during the postharvest storage phase in the Chiang Mai and Phitsanulok Provinces of northern Thailand. These diseases can lead to significant economic losses. This present study was conducted to isolate the causal agent of fungi in lesions of fruit rot. A total of four fungal isolates were obtained, of which two isolates (SDBR-CMU422 and SDBR-CMU423) were obtained from rot lesions of watermelons, while the remaining isolates (SDBR-CMU424 and SDBR-CMU425) were obtained from rot lesions of muskmelons. All fungal isolates were identified using both morphological characteristics and molecular analyses. Morphologically, all isolated fungal isolates were classified into the genus *Fusarium*. Multi-gene phylogenetic analyses of a combination of the translation elongation factor 1-alpha (*tef-1*), calmodulin (*cam*), and RNA polymerase second largest subunit (*rpb2*) genes reveled that four fungal isolates belonged to the *Fusarium incarnatum*–*equiseti* species complex and were distinct from all other known species. Thus, we have described them as two new species, namely *F. citrullicola* (SDBR-CMU422 and SDBR-CMU423) and *F. melonis* (SDBR-CMU424 and SDBR-CMU425). A full description, illustrations, and a phylogenetic tree indicating the position of both new species have been provided. Moreover, pathogenicity tests were subsequently performed and the results showed that *F. citrullicola* and *F. melonis* caused symptoms of fruit rot on inoculated watermelon and muskmelon fruits, respectively. Notably, this outcome was indicative of the symptoms that appeared during the postharvest storage phase. To our knowledge, two new pathogenic fungi, *F. citrullicola* and *F. melonis*, are new causal agents of watermelon and muskmelon fruit rot, respectively. Importantly, these findings provide valuable information for the development of effective strategies for the monitoring and prevention of these diseases.

## 1. Introduction

Watermelon [*Citrullus lanatus* (Thunb.) Mats. & Nakai] and muskmelon (*Cucumis melo* L.) are both cucurbit species that belong to the family Cucurbitaceae [1]. Both crops are extensively cultivated in temperate, subtropical, and tropical regions throughout the world [2,3]. Both fruits have been described as healthy food choices for human consumption [4,5]. The primary nutritional components found in watermelon and muskmelon fruits include amino acids, ascorbic acid, β-carotene, carbohydrates, fatty acids, flavonoids, minerals, potassium, sugars, vitamins, and a number of bioactive compounds [2,5,6,7,8]. They also possess beneficial medicinal properties such as analgesic, anticancer, anti-inflammatory, antimicrobial, antioxidant, antiulcer, and hepatoprotective properties [9,10]. More interestingly, the watermelon fruit is a great natural source of lycopene and also principally contains about 93% water [2]. In 2020, China is known to be the largest producer of watermelon and melon (including muskmelon) fruits in the global market, followed by Turkey, India, and Iran [11]. In Southeast Asia, Indonesia led in melon production in 2020, followed by the Lao People’s Democratic Republic and the Philippines [11]. In addition, Vietnam led Southeast Asia in watermelon production in 2020, followed by Indonesia, the Lao People’s Democratic Republic, and Thailand [11]. Currently, muskmelon has emerged as one of the economically important crops in Thailand.

Several diseases caused by microorganisms (bacteria, fungi, and viruses) can have negative effects on cucurbit plants throughout both the growing period and the postharvest period [4,12,13,14]. Fruit rot disease is one of the most typical preharvest and postharvest diseases of cucurbit fruits in Thailand and worldwide [4,15,16,17,18,19]. This disease has caused huge losses through reductions in harvest yields and lowered standards of quality, both of which can have a significant negative economic impact [20,21,22]. Previous studies indicate that the fungal species belonging to the genera *Alternaria*, *Didymella*, *Epicoccum*, *Fusarium*, *Lasiodiplodia*, *Myrothecium*, *Penicillium*, *Phomopsis*, *Phytophthora*, *Pythium*, *Rhizoctonia*, and *Sclerotium* have been reported as causal agents of fruit rot in cucurbits (cantaloupes, cucumbers, melons, pumpkins, squashes, and watermelons) [4,15,17,23,24,25,26]. The demand for watermelon and muskmelon fruits has risen due to the rapid growth of the world’s population and an increased interest in pursuing a healthy lifestyle. Therefore, plantation areas dedicated to the cultivation of both watermelon and other melon plants have increased significantly. Conversely, the prevalence and severity of some fungal-based diseases have also increased when plants have been grown in sub-optimal locations [4,27,28]. In Thailand, the major areas for watermelon and melon cultivation are located in the northern region of the country, including Chiang Mai, Kamphaeng Phet, Phayao, Phichit, Phitsanulok, and Sukhothai Provinces. However, there have been relatively few studies on incidences of postharvest fruit rot of watermelon and muskmelon in Thailand. In 2022, fruit rot caused by fungi was observed on watermelon and muskmelon during the postharvest storage phase in Chiang Mai and Phitsanulok Provinces, respectively. The incidence of this disease ranged from 20 to 30% according to the number of fruits in each pallet box (100 fruits per pallet box). Therefore, this study aimed to isolate the causal fungal agents of these fruit rot diseases. The obtained fungi were identified and described by their morphological characteristics and through multigene phylogenetic analysis. Subsequently, a pathogenicity test was subsequently performed and Koch’s postulates were employed to evaluate the asymptomatic fruits of watermelon and muskmelon using the isolated fungi.

## 2. Materials and Methods

### 2.1. Sample Collection

Fruit rot disease on watermelon (*C. lanatus*) and muskmelon (*C. melo*) fruits was observed during the postharvest storage phase in Chiang Mai and Phitsanulok Provinces of northern Thailand in 2022. Ten fruits of each symptomatic watermelon and muskmelon from different locations were randomly collected, maintained in sterile plastic boxes, and carried to the laboratory within 48 h of collection. After being transferred to the laboratory, symptomatic fruits were examined using a stereo microscope (Nikon H55OS, Tokyo, Japan) and then stored in a plastic box with wet filter paper in order to stimulate sporulation.

### 2.2. Fungal Isolation

Fruits samples were processed for the isolation of fungal causal agents. Causal fungi were isolated from lesions using a single conidial isolation on 1.0% water agar containing 0.5 mg/l streptomycin under a stereo microscope according to the method described by Choi et al. [29]. The isolated plates were incubated at 25 °C in darkness for 24–48 h, and the germinated conidia were then transferred onto potato dextrose agar (PDA; Conda, Madrid, Spain) containing 0.5 mg/L streptomycin. Pure fungal isolates were deposited in the culture collection of the Sustainable Development of Biological Resources, Faculty of Science, Chiang Mai University (SDBR-CMU), Chiang Mai Province, Thailand.

### 2.3. Fungal Identification

#### 2.3.1. Morphological Study

The morphological characteristics of fungal isolates were determined according to methods established by Crous et al. [30] and Wang et al. [31,32]. Colony characteristics, including colony morphology, pigmentation, and odor, were observed on PDA, oatmeal agar (OA; Difco, Le Pont de Claix, France), and synthetic nutrient-poor agar (SNA) after one week of incubation in the dark at 25 °C. Color notations were rated according to the color charts of Kornerup and Wanscher [33]. Micromorphological characteristics were identified using sterile water as a mounting medium under a light microscope (Nikon Eclipse Ni-U, Tokyo, Japan). Anatomical structure related to size data (e.g., chlamydospores, conidiophores, conidiogenous cells, conidia, and phialides) was based on at least 50 measurements of each structure using the Tarosoft (R) Image Frame Work program.

#### 2.3.2. DNA Extraction and PCR Amplification and Sequencing

Genomic DNA of each fungal isolate was extracted from the fungal mycelia grown on PDA in darkness at 25 °C for five days using the Fungal DNA Extraction Kit (FAVORGEN, Ping-Tung, Taiwan) following the manufacturer’s protocol. The translation elongation factor 1-alpha (*tef-1*), calmodulin (*cam*), and RNA polymerase second largest subunit (*rpb2*) genes were amplified by the polymerase chain reaction (PCR) using EF1/EF2 primers [34], CAL-228F/CAL-2Rd primers [35] and RPB2-5F2/RPB2-7cR primers [36], respectively (Table 1). The amplification program of three genes was conducted in separate PCR reactions. The amplification process consisted of an initial denaturation step at 95 °C for 3 min, followed by 35 cycles of denaturation at 95 °C for 30 s, annealing steps at 60 °C for 50 s (*tef-1*), 59 °C for 30 s (*cam*) and 52 °C for 1 min (*rpb2*), and a final extension step at 72 °C for 1 min on a peqSTAR thermal cycler (PEQLAB Ltd., Fareham, UK). PCR products were checked on 1% agarose gel electrophoresis.

#### 2.3.3. Sequencing

PCR products were purified using the PCR Clean-Up Gel Extraction NucleoSpin^®^ Gel (Macherey-Nagel, Düren, Germany) according to the manufacturer’s protocol. The purified PCR products were directly sequenced. Sequencing reactions were performed and the sequences were then automatically determined in a genetic analyzer at the 1st Base Company Co., Ltd., (Kembangan, Malaysia) using EF1/EF2, CAL-228F/CAL-2Rd and RPB2-5F2/RPB2-7cR primers for *tef-1*, *cam*, and *rpb2*, respectively.

#### 2.3.4. Sequence Alignment and Phylogenetic Analyses

An analysis of the *tef-1*, *cam*, and *rpb2* sequences was carried out with the use of similarity searches using the BLAST program available at NCBI (https://blast.ncbi.nlm.nih.gov, accessed on 5 August 2022). The sequences from this study, along with those obtained from previous studies and the GenBank database (with ≥60% query coverage and ≥85–100% sequence similarity) were selected and are listed in Table 2. Multiple sequence alignment was performed with MUSCLE [37] and improved where necessary using BioEdit v. 6.0.7 [38]. Phylogenetic analysis was carried out using combination datasets of *tef-1*, *cam*, and *rpb2*. *Fusarium camptoceras* CBS 193.65 and *F. neosemitectum* CBS 115476 within the *F. camptoceras* species complex (FCAMSC) were used as the outgroup. A phylogenetic tree was constructed under the maximum likelihood (ML) and Bayesian inference (BI) methods. The ML analysis was carried out using RAxML v7.0.3 on the GTRCAT model with 25 categories and 1000 bootstrap (BS) replications [39,40] via the online portal CIPRES Science Gateway v. 3.3 [41]. BI analysis was performed with MrBayes v3.2.6 software for Windows [42]. The best substitution models for BI and ML analyses were estimated using the jModelTest 2.1.10 [43] by employing the Akaike information criterion (AIC). Both ML and BI analyses were based on the GTR + I + G model. For BI analysis, six simultaneous Markov chains were run for one million generations with random initial trees, wherein every 1000 generations were sampled. A burn-in phase was employed to discard the first 2000 trees, while the remaining trees were used to construct the 50% majority-rule consensus phylogram with calculated Bayesian posterior probabilities (PP). The tree topologies were visualized in FigTree v1.4.0 [44].

### 2.4. Pathogenicity Tests

Asymptomatic commercial watermelon and muskmelon fruits were carefully washed, and the surfaces were disinfected by immersion in 1.5% (*v/v*) sterile sodium hypochlorite solution for 5 min. They were then subsequently washed three times with sterile distilled water. The surface disinfected fruits were then air-dried at room temperature (25 ± 2 °C) for 10 min [50]. After being air-dried, a uniform wound (5 pores, 1 cm in depth and 1 mm in width) was made at the equator of each fruit using aseptic needles [4]. Conidial suspensions of all fungal isolates were prepared from each fungal culture grown on PDA at 25 °C for two weeks and suspended in sterile distilled water. The suspension was filtered through two layers of sterile cheesecloth, diluted in distilled water with 0.05% (*v/v*) Tween 20, and adjusted to 1 × 10^6^ conidia/mL using a hemacytometer. Five hundred microliters of the conidial suspension was dropped onto the wounded fruits. Accordingly, control fruits were also wounded and treated with sterile distilled water. Each fruit was then placed in a separate sterile plastic box (26 cm × 35.5 cm × 20 cm) at conditions of 80% relative humidity. The plastic boxes were stored in a growth chamber at 25 °C under a 12 h period of light for one week. Ten replications were conducted for each treatment. The experiments were independently repeated twice. The disease severity score was employed to evaluate the specimens following the method described by Safari et al. [51] with mild (1–25%), moderate (26–50%), severe (51–75%), and very severe (76–100%) degrees of infection for the damaged fruit areas. To authenticate the causal agent, the fungi were re-isolated from the lesions following the method described by Bika and Baysal-Gurel [52].

## 3. Results

### 3.1. Sample Collection and Disease Symptoms

**Figure 1:**
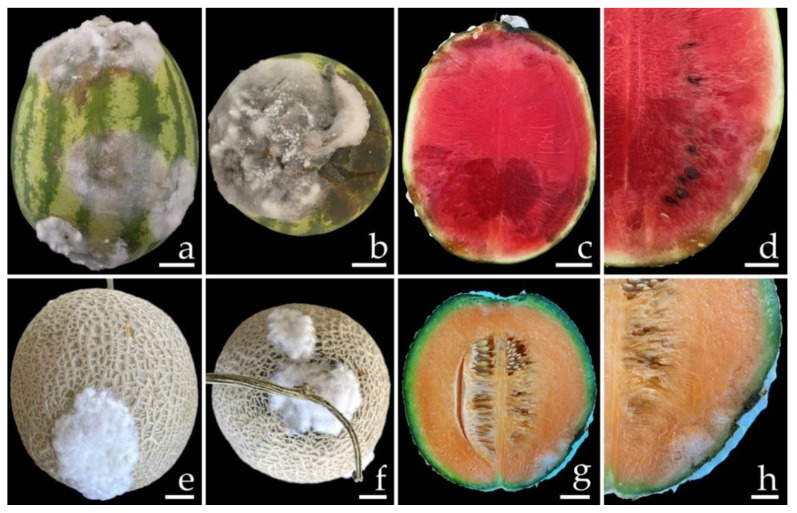
Natural symptoms of fruit rot disease on watermelon (**a**–**d**) and melon (**e**–**h**). The infected watermelon (**a**) and melon (**e**) fruits covered with white mycelium in the epidermal tissue. The top view of infected watermelon (**b**) and melon (**f**) fruits. A cross-section of a mature lesion of infected watermelon (**c**,**d**) and melon (**g**,**h**) fruits revealed the internal decayed area. Scale bars: (**a**–**c**) = 30 mm; (**d**) = 15 mm; (**e**–**g**) = 20 mm; (**h**) = 10 mm.

Samples of fruit rot on specimens of watermelon (*C. lanatus*) and muskmelon (*C. melo*) were collected from postharvest storage pallet boxes located in Chiang Mai and Phitsanulok Provinces of northern Thailand, respectively. The incidence of this disease ranged from 20 to 30% according to the number of fruits in each pallet box (100 fruits per pallet box). Symptoms on watermelon were characterized by the initial presence of small light-brown spots. These spots then expanded into irregular brown spots, and the epidermal tissue was covered with white mycelia tissue (Figure 1a,b). Disease symptoms on the muskmelon started at the top and base of the fruit appearing as brown spots surrounded by a bruise margin. Eventually, white mycelial masses covered the advanced lesions (Figure 1e,f). Lesions of both the watermelon and muskmelon fruit finally became widened and merged to cover the entire fruit, causing both of the infected fruits to appear bruised, ruptured, and decayed. The internal area of decay appeared to be clearly rotten and was surrounded by water-soaked tissue (Figure 1c,d,g,h).

### 3.2. Fungal Isolation

A total of four fungal isolates were obtained in this study. Two fungal isolates, CMU422 and CMU423, were isolated from watermelon fruits rot collected from Chiang Mai Province and two isolates, CMU424 and CMU425, were isolated from muskmelon fruits rot collected from Phitsanulok Province. All fungal isolates were deposited at the SDBR-CMU under the accession numbers SDBR-CMU422, SDBR-CMU423, SDBR-CMU424, and SDBR-CMU425, respectively.

### 3.3. Morphological Study

Fungal colonies of each isolate were observed on three different agar media including PDA, OA, and SNA at 25 °C. After being incubated for one week, OA was found to be the best media by displaying the highest colony diameter of all four isolates. All four fungal isolates produced conidiophores, conidiogenous cells, chlamydospores, phialides, and conidia in all of the agar media. Based on the morphological characteristics, all fungal isolates were initially identified as belonging to the genus *Fusarium* [30,31,32,45]. The results obtained from morphological observation of the fungal colony and the micromorphological characters revealed that the isolate SDBR-CMU422 was similar to the isolate SDBR-CMU423, and that the isolate SDBR-CMU424 was similar to the isolate SDBR-CMU425. The fungal identification was then further confirmed through multi-gene phylogenetic analysis of a combination of the translation elongation factor 1-alpha (*tef-1*), calmodulin (*cam*), and the RNA polymerase second largest subunit (*rpb2*) genes.

### 3.4. Phylogenetic Results

**Figure 2:**
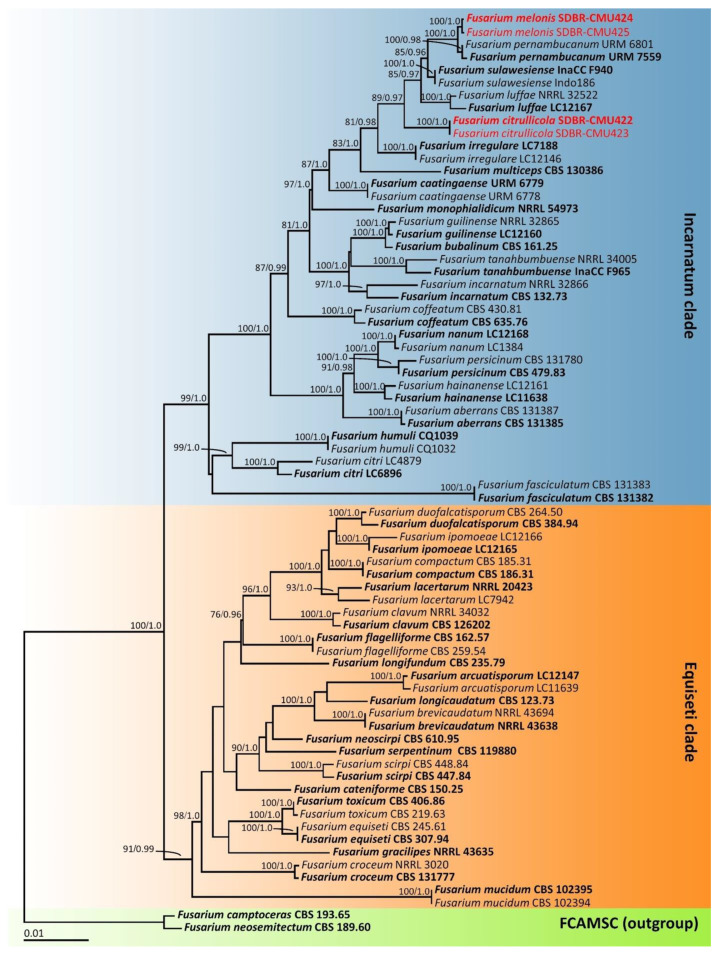
Phylogram derived from maximum likelihood analysis of 73 taxa of the combined *tef-1*, *cam*, and *rpb2* sequences. *Fusarium camptoceras* CBS 193.65 and *F. neosemitectum* CBS 115476 were used as the outgroup. The numbers above branches represent bootstrap percentages (**left**) and Bayesian posterior probabilities (**right**). Bootstrap values ≥ 75% and Bayesian posterior probabilities ≥ 0.95 are shown. The scale bar represents the expected number of nucleotide substitutions per site. Sequences of fungal species obtained in this study are in red. Type species are in bold.

The *tef-1*, *cam*, and *rpb2* sequences of each fungal isolate were amplified, sequenced, and deposited in the GenBank database (Table 2). The combined *tef-1*, *cam*, and *rpb2* sequence dataset consisted of 73 taxa, while the aligned dataset was comprised of 2096 characters including gaps (*tef-1*: 1–669, *cam*: 670–1231 and *rpb2*: 1232–2096). ML analysis of the combined dataset yielded a best scoring tree with a final ML optimization likelihood value of −9306.5763. The matrix contained 555 distinct alignment patterns with 5.24% undetermined characters or gaps. Estimated base frequencies were recorded as follows: A = 0.2810, C = 0.2397, G = 0.2704, T = 0.2089; while substitution rates were established as AC = 0.6704, AG = 5.9889, AT = 0.7165, CG = 0.8948, CT = 19.6030, GT = 1.0000. The gamma distribution shape parameter alpha value was equal to 0.2311 and the tree-length value was equal to 0.6021. In addition, the final average standard deviation of the split frequencies at the end of the total MCMC generations was calculated as 0.00708 through BI analysis. In terms of topology, the phylograms of the ML and BI analyses were found to be similar (data not shown). Therefore, the phylogram obtained from the ML analysis was selected and is presented in Figure 2. Our phylogenetic tree was constructed concordantly and is supported by previous studies [30,31,32,45]. A phylogram clearly separated the four fungal isolates obtained in this study into two monophyletic clades within the *Incarnatum* clade of the *Fusarium incarnatum*–*equiseti* species complex. The results indicate that the sequences of two fungal isolates, SDBR-CMU422 and SDBR-CMU423 (introduced as *F. citrullicola*), were clearly separated from the previously known *Fusarium* species in the *Incarnatum* clade with a high support value (100% BS and 1.0 PP). Moreover, two fungal isolates, SDBR-CMU424 and SDBR-CMU425 (introduced as *F. melonis*), formed a sister taxon to *F. pernambucanum* with high BS (100%) and PP (1.0) supports.

### 3.5. Taxonomic Description

**Figure 3:**
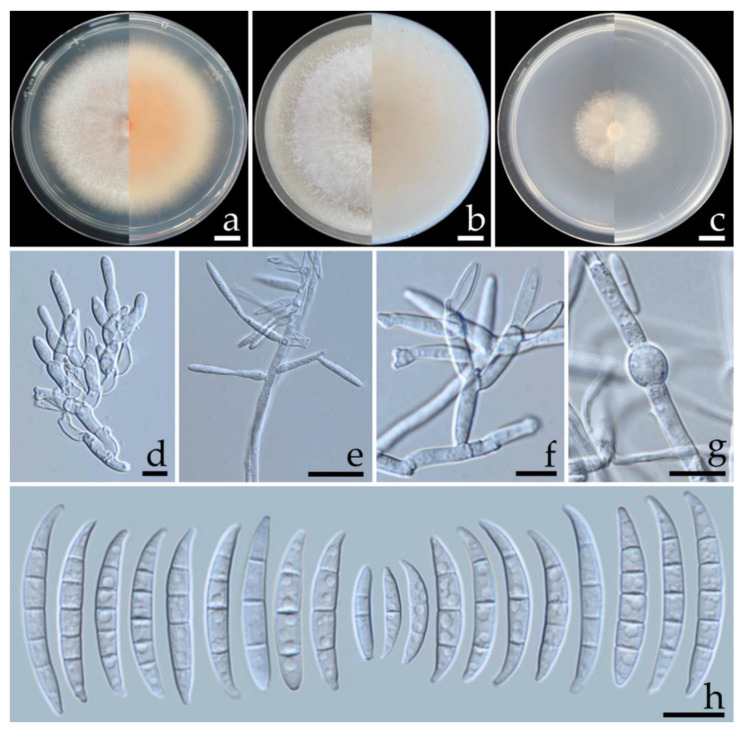
***Fusarium citrullicola*** (SDBR-CMUS422; holotype). Colonies incubated at 25 °C for one week on PDA (**a**), OA (**b**), and SNA (**c**) (left, surface view and right, reverse view). Conidiophores on aerial mycelium (**d**). Lateral monophialides on aerial mycelium (**e**). Polyphialides on aerial mycelium (**f**). Chlamydospores (**g**). Aerial conidia (**h**). Scale bars: (**a**–**c**) = 10 mm; (**d**–**h**) = 10 µm.

***Fusarium citrullicola*** S. Khuna, J. Kumla & N. Suwannarach, sp. nov. (Figure 3).

MycoBank No.: 845955.

Etymology: ‘*citrullicola’* referring to the *Citrullus*-inhibitor.

Holotype: THAILAND, Chiang Mai Province, Mueang District, 18°45′31″N, 98°58′20″E, on fruit rot lesion of *Citrullus lanatus*, 18 May 2022, S. Khuna, ex-type culture: SDBR-CMU422.

Description: Colonies on PDA, OA, and SNA were described at 25 °C after seven days of incubation. Colonies on PDA grew to 68.0–74.5 mm in diameter, slightly raised, aerial mycelia dense, colony margin entire, orange white (6A2) in the center, white at the margin; reverse light orange (6A5) in the center, white at the margin. Colonies on OA reached 75.0–85.0 mm in diameter, umbilicate, aerial mycelia dense, colony margin entire, surface white; reverse pale orange (5A3) in the center, white at the margin. Colonies on SNA attained a diameter of 45.5–51.0 mm, flat, aerial mycelia scant, colony margin entire, surface white; reverse white. Pigment and odor absent. No sporodochia were observed in all agar media. Conidiophores borne on aerial mycelium, 10–120 × 1.8–3.2 µm, unbranched, sympodial, or irregularly branched, bearing terminal or lateral phialides, with apical whorls of 1–3 phialides. Phialides mono- and polyphialidic, subulate to subcylindrical, sometimes proliferating percurrently, smooth and thin-walled, hyaline, 8.4–30.4 × 2.0–4.7 µm (av. ± SD: 16.8 ± 5.3 × 2.9 ± 0.5 µm). Chlamydospores abundant, intercalarily or terminal, globose, ellipsoid, smooth, thick-walled, hyaline, 0–4-septate, 4.7–15.6 × 4.6–14.4 µm (av. ± SD: 10.0 ± 2.7 × 7.7 ± 2.5 µm). Conidia falcate, curved dorsiventrally, sometimes straight, tapering towards both ends, smooth to slightly rough, hyaline, apical cell pointed to blunt, basal cell blunt to barely notched, 1–5-septate; 1-septate conidia 8.0–21.0 × 2.0–3.8 µm (av. ± SD: 14.9 ± 2.7 × 2.8 ± 0.4 µm); 2-septate conidia 13.9–24.6 × 2.1–3.9 µm (av. ± SD: 19.3 ± 2.4 × 3.1 ± 0.4 µm); 3-septate conidia 17.7–34.4 × 2.3–3.9 µm (av. ± SD: 26.6 ± 3.8 × 3.1 ± 0.4 µm); 4-septate conidia 26.7–35.3 × 2.4–4.4 µm (av. ± SD: 31.0 ± 2.3 × 3.7 ± 0.5 µm); 5-septate conidia 28.0–39.0 × 2.4–4.9 µm (av. ± SD: 32.7 ± 2.4 × 3.9 ± 0.4 µm).

Additional specimen examined: THAILAND, Chiang Mai Province, Mueang District, 18°45′31″N, 98°58′20″E, on fruit rot lesion of *Citrullus lanatus*, 18 May 2022, S. Khuna, SDBR-CMU423.

GenBank accession numbers: holotype SDBR-CMUS422 (*tef-1*: OP020920, *cam*: OP020924, *rpb2*: OP020928); additional specimen SDBR-CMUS423 (*tef-1*: OP020921, *cam*: OP020925, *rpb2*: OP020929).

Note: The colony characteristics of *F. citrullicola* on PDA were similar to those of *F. sulawesiense* that were found to have caused crown rot on banana fruit [31,49] and black rot on papaya fruit [53]. However, the growth of *F. citrullicola* displayed faster growth than *F. sulawesiense* (36.4–42.0 mm) on PDA at 25 °C [49]. The phylogenetic analyses of the combined *tef-1*, *cam*, and *rpb2* sequences confirmed that *F. citrullicola* was clearly distinguishable from *F. sulawesiense* and the previously known *Fusarium* species in the *Incarnatum* clade with a high support value (Figure 2).

**Figure 4:**
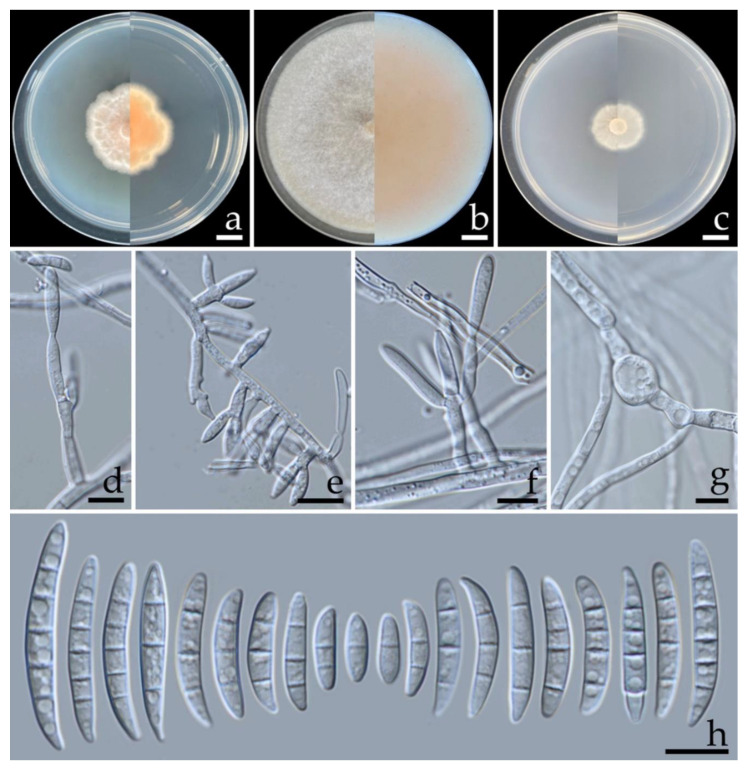
***Fusarium melonis*** (SDBR-CMUS424; holotype). Colonies incubated at 25 °C for one week on PDA (**a**), OA (**b**), and SNA (**c**) (left, surface view and right, reverse view). Conidiophores on aerial mycelium (**d**). Mono- and polyphialides on aerial mycelium (**e**). Polyphialides on aerial mycelium (**f**). Chlamydospores (**g**). Aerial conidia (**h**). Scale bars: (**a**–**c**) = 10 mm; (**d**–**h**) = 10 µm.

***Fusarium melonis*** S. Khuna, J. Kumla & N. Suwannarach, sp. nov. (Figure 4).

MycoBank No.: 845956.

Etymology: ‘*melonis’* referring to the host plant, *Cucumis melo*.

Holotype: THAILAND, Phitsanulok Province, Wang Thong District, 16°50′37″N, 100°36′00″E, on fruit rot lesion of *Cucumis melo*, 17 March 2022, S. Khuna, ex-type culture: SDBR-CMU424.

Description: Colonies on PDA, OA, and SNA were described at 25 °C after seven days of incubation. Colonies on PDA were 32.5–38.0 mm in diameter, flat, aerial mycelia scant, colony margin undulate, light orange (6A4) in the center, white at the margin; reverse orange (6A7) in the center, white at the margin. Colonies on OA grew to 85.0 mm in diameter, flat, aerial mycelia dense, colony margin entire, surface white; reverse light orange (6A4) in the center, white at the margin. Colonies on SNA reached a diameter of 15.0–20.5 mm, flat, aerial mycelia scant, colony margin entire, white; reverse white. Pigment and odor absent. No Sporodochia were observed in all agar media. Conidiophores borne on aerial mycelium, 13–85 × 1.9–4.2 µm, unbranched, sympodial branched, bearing terminal or lateral phialides, with apical whorls of 1–3 phialides. Phialides mono- and polyphialidic, subulate to sub-cylindrical, smooth and thin-walled, hyaline, 10.2–35.3 × 2.3–3.8 µm (av. ± SD: 18.6 ± 5.2 × 3.0 ± 0.4 µm). Chlamydospores abundant, intercalarily or terminal, globose, ellipsoid, smooth, thick-walled, hyaline, 0–2-septate, 5.3–15.3 × 4.7–12.8 µm (av. ± SD: 9.5 ± 2.2 × 7.9 ± 1.9 µm). Conidia ellipsoidal to falcate, slightly curved, sometimes straight, smooth to slightly rough, hyaline, apical cell pointed to blunt, basal cell obtuse to papillate, non-foot shaped, 1–5-septate; 1-septate conidia 11.7–22.8 × 2.6–4.0 µm (av. ± SD: 16.4 ± 2.1 × 3.2 ± 0.3 µm); 2-septate conidia 15.6–23.4 × 2.9–4.3 µm (av. ± SD: 18.8 ± 1.6 × 3.5 ± 0.3 µm); 3-septate conidia 17.9–31.3 × 3.1–4.6 µm (av. ± SD: 25.9 ± 3.2 × 3.6 ± 0.3 µm); 4-septate conidia 26.2–34.0 × 3.2–4.6 µm (av. ± SD: 30.4 ± 1.6 × 3.8 ± 0.3 µm); 5-septate conidia 26.7–45.8 × 3.2–4.8 µm (av. ± SD: 33.6 ± 4.0 × 4.0 ± 0.4 µm).

Additional specimen examined: THAILAND, Phitsanulok Province, Wang Thong District, 16°50′37″N, 100°36′00″E, on fruit rot lesion of *Cucumis melo*, 17 March 2022, S. Khuna, SDBR-CMU425.

GenBank accession numbers: holotype SDBR-CMUS424 (*tef-1*: OP020922, *cam*: OP020926, *rpb2*: OP020930); additional specimen SDBR-CMUS425 (*tef-1*: OP020923, *cam*: OP020927, *rpb2*: OP020931).

Note: Morphologically, *F. melonis* was similar to *F. pernambucanum*. However, the colony diameter of *F. melonis* on PDA at 25 °C (32.5–38.0 mm in diameter) was clearly smaller than *F. pernambucanum* (52.5–84 mm in diameter) [47]. The multi-gene phylogenetic analyses indicated that *F. melonis* clearly distinguished it from the other previously known *F. incarnatum–equiseti* species complexes and formed a sister clade to *F. pernambucanum*. However, a pairwise nucleotide comparison of *tef-1* data also indicated that *F. melonis* differed from *F. pernambucanum* by 2.2% (15/690 bp). Furthermore, *F. pernambucanum* was isolated from fruit rot disease of muskmelons (*C. melo*) grown in China [54], fruit rot disease on melons collected from Brazil [55], leaf blight disease found on plum trees (*Prunus salicina*) grown in China [56] and insects (*Aleurocanthus woglumi* and *Dactylopius opuntiae*) indigenous to Brazil [47].

### 3.6. Pathogenicity Test

**Figure 5:**
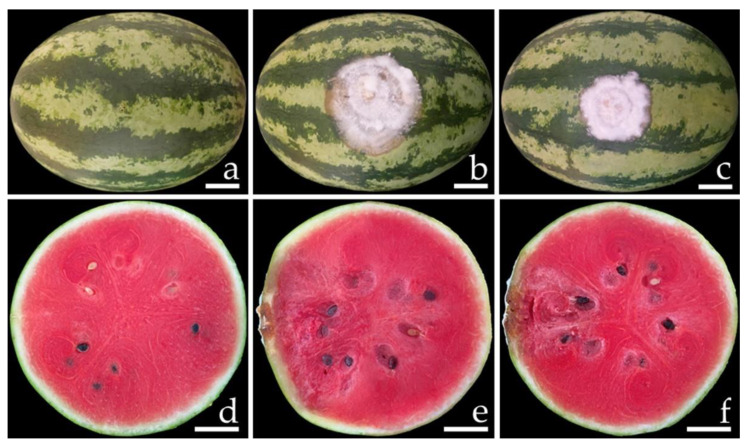
Pathogenicity test using *F. citrullicola* SDBR-CMU422 and SDBR-CMU423 on watermelon (*Citrullus lanatus*) fruits after one week of inoculation. Control fruit treated with water instead of inoculum (**a**,**d**). Fruit rot after inoculation of isolate SDBR-CMU422 (**b**,**e**) and isolate SDBR-CMU423 (**c**,**f**). Scale bars = 30 mm.

**Figure 6:**
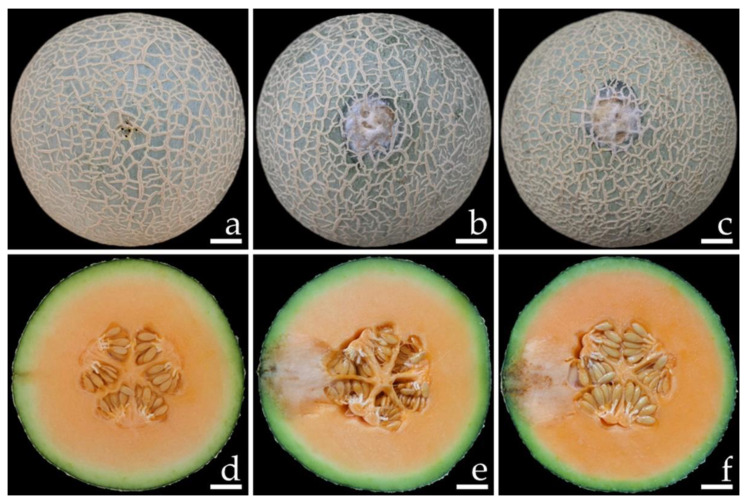
Pathogenicity test using *F. melonis* SDBR-CMU424 and SDBR-CMU425 on melon (*Cucumis melo*) fruits after one week of inoculation. Control fruit treated with water instead of inoculum (**a**,**d**). Fruit rot after inoculation of isolate SDBR-CMU424 (**b**,**e**) and isolate SDBR-CMU425 (**c**,**f**). Scale bars = 20 mm.

Conidia of all fungal isolates were used in this experiment. The initial symptoms were observed on both inoculated watermelon and muskmelon fruits two days after inoculation. Initially, small light-brown to brown spots appeared on the fruits. The lesions then enlarged rapidly and developed into brown to dark brown spots on the watermelon fruit and green bruised spots on the melon fruit, both of which were covered with white mycelia surrounding each lesion. After one week of incubation, the lesion diameters on the inoculated fruits were within the ranges of 6.0–6.5 and 2.5–3.0 cm on the watermelons (Figure 5b,c) and muskmelons (Figure 6b,c), respectively. The inoculated watermelons exhibited moderate infections indicated by symptoms of rot, whereas the muskmelons exhibited mild infections. A cross-section revealed that the internal lesion area appeared to be rotting and was surrounded by water-soaked tissue (Figure 5e,f and 6e,f). The diameters of the internal lesions on the watermelon and muskmelon fruits ranged from 6.5–7.5 and 4.0–4.5 cm, respectively. The lesions then spread to the entire fruit and coalesced within 12 and 14 days on the watermelon and muskmelon specimens, respectively, after the occurrence of necrosis. After that, the fruits became completely soft and rotten. These disease symptoms were similar to those seen during the postharvest storage phase. However, no symptoms of plant disease were observed in the inoculation treatments involving sterile distilled water among both wounded watermelon (Figure 5a,d) and muskmelon (Figure 6a,d) fruits. The fungi were re-isolated from symptomatic fruit tissue and then cultured on PDA in order to fulfill Koch’s postulates. The re-isolated fungi were identified as *F. citrullicola* and *F. melonis*.

## 4. Discussion

With regard to the genus *Fusarium* (Nectriaceae, Hypocreales), currently, there are more than 400 accepted species that have been divided into 23 species complexes [32,57]. The *Fusarium* species is known to cause several diseases including crown rot, root rot, wilt, fruit rot, leaf spot, stem rot, and seedling blight among cucurbits (cucumbers, muskmelons, pumpkins, squash, and watermelons) worldwide [14,15,23,54,58,59,60]. Traditionally, *Fusarium* species are mainly identified by macromorphological characteristics (colony morphology, pigmentation, and type of aerial mycelium), and micromorphological characteristics (the shapes and sizes of conidiophores, conidiogenous cells, macroconidia, microconidia, and the presence or absence of chlamydospores) [30,61,62]. However, morphological characteristics cannot be used to distinguish between the closely related species of *Fusarium* due to the wide range of morphological variations [30,61]. Therefore, it is essential to identify *Fusarium* species by applying a molecular approach. Ribosomal DNA [the internal transcribed spacer (ITS) and the large subunit (LSU) regions] and protein-coding [*cam*, *tef-1*, β-tubulin (*tub2*), RNA polymerase largest subunit (*rpb1* and *rpb2*)] genes have provided researchers with a powerful tool in the identification of the *Fusarium* species [30,31,36,49,63,64,65]. Nevertheless, using only the ribosomal DNA gene did not resolve the identification of *Fusarium* at the species level [66,67]. Currently, a combination of morphological characteristics and multi-gene molecular phylogeny are being used for the accurate identification of the *Fusarium* species [30,31,32,45,47,36,49,63,64,65]. In this study, two new *Fusarium* species, namely *F. citrullicola* and *F. melonis*, were obtained from the rot lesions of watermelon and muskmelon fruits, respectively, that were collected from northern Thailand. All fungal species were identified according to their morphological and molecular characteristics in accordance with the identification methods established for the identification approach of *Fusarium* [30,31,32,47].

Both of our new fungal species belong to the *F. incarnatum–equiseti* species complex (FIESC). FIESC is a highly diverse group that is widely distributed. Accordingly, the majority of them are saprobes that are recognized as pathogens of plants, humans, and animals, and have been found in various environmental habitats [4,17,18,19,31,46,68,69]. Generally, identification based only on the morphological characteristics of FIESC is difficult because many species have similar outward appearances and display overlapping microscopic characteristics [61,70]. Therefore, molecular multi-gene phylogenetic analysis would be essential to accurately identify the FIESC species [31,45]. Prior to this study, this species complex comprised of 38 recognized phylogenetic species and has been separated into two main clades including the *Equiseti* clade (19 species) and the *Incarnatum* clade (19 species) [31,45,47,65,70]. Our multi-gene phylogenetic analyses revealed that two new species, *F. citrullicola* and *F. melonis*, formed distinct lineages from previously known species within the *Incarnatum* clade of FIESC. The different colony characteristics between the two new species indicate that *F. citrullicola* could more effectively grow on PDA and SNA when compared with *F. melonis*. On the OA medium, *F. citrullicola* generated an umbilicate colony, but *F. melonis* established a flat colony. Micromorphological characteristics indicate that *F. citrullicola* presents falcate conidia with tapering towards both ends, while *F. melonis* presents ellipsoidal to falcate conidia that are slightly curved. Additionally, *F. citrullicola* presents four septate chlamydospores, whereas *F. melonis* presents two septate chlamydospores. Phylogenetic analyses confirmed that *F. citrullicola* and *F. melonis* are actually different species. In addition, *F. melonis* formed a sister clade to *F. pernambucanum*. However, a nucleotide comparison of the *tef-1* gene showed that *F. melonis* differed from *F. pernambucanum* by 2.2% (15/690 bp). Jeewon and Hyde [71] suggested that the nucleotide comparisons of reliable genes should be more than 1.5% different in order to justify the existence of a novel species. Therefore, *F. melonis* and *F. pernambucanum* can be considered different species.

To fulfill Koch’s postulates, pathogenicity tests were conducted on all isolates of *F. citrullicola* and *F. melonis* that had manifested the same symptoms as those observed during the postharvest storage phase. Therefore, *F. citrullicola* and *F. melonis* can be considered causal agents for watermelon and muskmelon fruit rot, respectively. Our results are supported by the findings of several previous studies, which indicated that *Fusarium* is an economically significant plant pathogen. Accordingly, some species of FIESC have been reported to cause fruit rot disease in various cucurbit plants worldwide [4,18]. For example, *F. equiseti* caused fruit rot disease on watermelon specimens collected in China [72], Malaysia [18], and the United States [24]. Ezrari et al. [16] found that *F. equiseti* caused pre- and postharvest fruit rot on zucchini plants (*Cucurbita pepo*) in Morocco. Notably, *F. equiseti* has been reported as a causal agent of postharvest fruit rot on both oriental melons and cantaloupes grown in Korea [73] and Thailand [4], respectively. *Fusarium incarnatum* was found as a causal agent of fruit rot on cucumbers grown in Mexico [17] and muskmelons cultivated in Thailand [19]. *Fusarium pernambucanum* caused fruit rot disease on muskmelons grown in China [54]. In Brazil, *F. pernambucanum* and *F. sulawesiense* were found to cause fruit rot on melons [55]. Additionally, other *Fusarium* species in the *F. chlamydosporum* species complex (*F. chlamydosporum*), the *F. fujikuroi* species complex (*F. annulatum*, *F. moniliforme*, *F. proliferatum*, and *F. verticillioides*), the *F. solani* species complex (*F. falciforme*, *F. petroliphilum*, and *F. solani*), the *F. oxysporum* species complex (*F. kalimantanense*), the *F. sambucinum* species complex (*F. asiaticum*, *F. culmorum*, *F. graminearum*, and *F. sambucinum*) and the *F. tricinctum* species complex (*F. acuminatum*) were also found to cause fruit rot on numerous cucurbits (cucumbers, melons, pumpkins, squashes, and watermelons) [22,25,55,73,74,75,76,77,78,79].

In Thailand, *F. equiseti* and *F. incarnatum* were found to be causal agents of rot among cantaloupes [4] and muskmelons [19], respectively. In addition, *F. equiseti* has been reported as a causal agent of muskmelon wilt disease [80]. In the current study, the disease symptoms observed in incidences of watermelon and muskmelon fruit rot caused by *F. citrullicola* and *F. melonis*, respectively, are similar to those that were caused by those known pathogens. Therefore, we have proposed that *F. citrullicola* and *F. melonis* be added as the causal agents of fruit rot on watermelons and muskmelons, respectively. However, there have been no prior reports of fruit rot disease on watermelons grown in Thailand. Thus, this study was determined to be the first investigative report on watermelon fruit rot in Thailand. Generally, watermelons and muskmelons are cultivated and harvested in Thailand throughout the cool to early wet seasons (November to June). Thus, during these seasons, fruit rot in watermelons and muskmelons can be found. Follow-up studies are needed to clarify the timing of the infections that occur in these fruits via fungal pathogens. This can be accomplished by monitoring the presence of the disease causal agents in these fruits at different stages of development in cultivation areas during both the pre-and postharvest processes, as well as during the postharvest storage period. Additional investigations will also be necessary to determine the disease’s inoculum source and the meteorological conditions that influence infection and disease development.

## 5. Conclusions

Fruit rot disease caused by the *Fusarium* species is one of the most important postharvest diseases of cucurbits in the world. In this study, two new pathogenic *Fusarium* species, namely *F. citrullicola* and *F. melonis*, were isolated from infected watermelon and melon fruits, respectively. Their identification was based on morphological characteristics and multi-gene phylogenetic analyses. The pathogenicity test of *F. citrullicola* and *F. melonis* revealed the same symptoms under artificial inoculation conditions as those observed during the postharvest storage phase. Thus, *F. citrullicola* and *F. melonis* have been proposed as new causal agents of watermelon and muskmelon fruit rot, respectively. Consequently, further studies involving the distribution of these diseases in other regions of Thailand, and the control of these diseases, will need to be conducted. In order to address the significant economic losses caused by this disease, it will be essential to develop effective monitoring and preventative strategies in the future.

## Figures and Tables

**Table 1:** Details of primers and the obtained PCR product in this study.

Gene	Primer Name	Primer Sequence	The Obtained Length (bp)			
			SDBR-CMU422	SDBR-CMU423	SDBR-CMU424	SDBR-CMU425
*tef-1*	EF1 EF2	5′-ATGGGTAAGGARGACAAGAC-3′ 5′-GGARGTACCAGTSATCATG-3′	692	691	691	686
*cam*	CAL-228F CAL-2Rd	5′-GAGTTCAAGGAGGCCTTCTCCC-3′ 5′-TGRTCNGCCTCDCGGATCATCTC-3′	606	603	601	597
*rpb2*	RPB2-5F2 RPB2-7cR	5′-GGGGWGAYCAGAAGAAGGC-3′ 5′-CCCATRGCTTGYTTRCCCAT-3′	1152	1148	1141	1131

**Table 2:** Details of sequences used in the molecular phylogenetic analysis.

Fungal Taxa	Strain/Isolate	GenBank Accession Number			Reference
		*tef-1*	*cam*	*rpb2*	
*Fusarium aberrans*	CBS 131385 ^T^	MN170445	MN170311	MN170378	[45]
*F. aberrans*	CBS 131387	MN170446	MN170312	MN170379	[45]
*F. arcuatisporum*	LC12147 ^T^	MK289584	MK289697	MK289739	[31]
*F. arcuatisporum*	LC11639	MK289586	MK289658	MK289736	[31]
*F. brevicaudatum*	NRRL 43638 ^T^	GQ505665	GQ505576	GQ505843	[46]
*F. brevicaudatum*	NRRL 43694	GQ505668	GQ505579	GQ505846	[46]
*F. bubalinum*	CBS 161.25 ^T^	MN170448	MN170314	MN170381	[45]
*F. caatingaense*	URM 6779 ^T^	LS398466	–	LS398495	[47]
*F. caatingaense*	URM 6778	LS398465	–	LS398494	[47]
*F. cateniforme*	CBS 150.25 ^T^	MN170451	MN170317	MN170384	[45]
*F. citri*	LC6896 ^T^	MK289617	MK289668	MK289771	[31]
*F. citri*	LC4879	MK289615	MK289665	MK289768	[31]
*F. citrullicola*	SDBR-CMU422 ^T^	OP020920	OP020924	OP020928	This study
*F. citrullicola*	SDBR-CMU423	OP020921	OP020925	OP020929	This study
*F. clavum*	CBS 126202 ^T^	MN170456	MN170322	MN170389	[45]
*F. clavum*	NRRL 34032	GQ505635	GQ505547	GQ505813	[46]
*F. coffeatum*	CBS 635.76 ^T^	MN120755	MN120696	MN120736	[48]
*F. coffeatum*	CBS 430.81	MN120756	MN120697	MN120737	[48]
*F. compactum*	CBS 186.31 ^ET^	GQ505648	GQ505560	GQ505826	[46]
*F. compactum*	CBS 185.31	GQ505646	GQ505558	GQ505824	[46]
*F. croceum*	CBS 131777 ^T^	MN170463	MN170329	MN170396	[45]
*F. croceum*	NRRL 3020	GQ505586	GQ505498	GQ505764	[46]
*F. duofalcatisporum*	CBS 384.94 ^T^	GQ505652	GQ505564	GQ505830	[46]
*F. duofalcatisporum*	CBS 264.50	GQ505651	GQ505563	GQ505829	[46]
*F. equiseti*	CBS 307.94 ^NT^	GQ505599	GQ505511	GQ505777	[46]
*F. equiseti*	CBS 245.61	GQ505594	GQ505506	GQ505772	[46]
*F. fasciculatum*	CBS 131382 ^T^	MN170473	MN170339	MN170406	[45]
*F. fasciculatum*	CBS 131383	MN170474	MN170340	MN170407	[45]
*F. flagelliforme*	CBS 162.57 ^T^	GQ505645	GQ505557	GQ505823	[46]
*F. flagelliforme*	CBS 259.54	GQ505650	GQ505562	GQ505828	[46]
*F. gracilipes*	NRRL 43635 ^T^	GQ505662	GQ505573	GQ505840	[46]
*F. guilinense*	LC12160 ^T^	MK289594	MK289652	MK289747	[31]
*F. guilinense*	NRRL 32865	GQ505614	GQ505526	GQ505792	[46]
*F. hainanense*	LC11638 ^T^	MK289581	MK289657	MK289735	[31]
*F. hainanense*	LC12161	MK289595	MK289648	MK289748	[31]
*F. humuli*	CQ1039 ^T^	MK289570	MK289712	MK289724	[31]
*F. humuli*	CQ1032	MK289568	MK289710	MK289722	[31]
*F. incarnatum*	CBS 132.73 ^NT^	MN170476	MN170342	MN170409	[45]
*F. incarnatum*	NRRL 32866	GQ505615	GQ505527	GQ505793	[46]
*F. ipomoeae*	LC12165 ^T^	MK289599	MK289704	MK289752	[31]
*F. ipomoeae*	LC12166	MK289600	MK289706	MK289753	[31]
*F. irregulare*	LC7188 ^T^	MK289629	MK289680	MK289783	[31]
*F. irregulare*	LC12146	MK289583	MK289682	MK289738	[31]
*F. lacertarum*	NRRL 20423 ^T^	GQ505593	GQ505505	GQ505771	[46]
*F. lacertarum*	LC7942	MK289643	MK289696	MK289797	[31]
*F. longicaudatum*	CBS 123.73 ^T^	MN170481	MN170347	MN170414	[45]
*F. longifundum*	CBS 235.79 ^T^	GQ505649	GQ505561	GQ505827	[46]
*F. luffae*	LC12167 ^T^	MK289601	MK289698	MK289754	[31]
*F. luffae*	NRRL 32522	GQ505612	GQ505524	GQ505790	[46]
*F. melonis*	SDBR-CMU424 ^T^	OP020922	OP020926	OP020930	This study
*F. melonis*	SDBR-CMU425	OP020923	OP020927	OP020931	This study
*F. monophialidicum*	NRRL 54973 ^T^	MN170483	MN170349	MN170416	[45]
*F. mucidum*	CBS 102395 ^T^	MN170485	MN170351	MN170418	[45]
*F. mucidum*	CBS 102394	MN170484	MN170350	MN170417	[45]
*F. multiceps*	CBS 130386 ^T^	GQ505666	GQ505577	GQ505844	[46]
*F. nanum*	LC12168 ^T^	MK289602	MK289651	MK289755	[31]
*F. nanum*	LC1384	MK289611	MK289661	MK289764	[31]
*F. neoscirpi*	CBS 610.95 ^T^	GQ505601	GQ505513	GQ505779	[46]
*F. pernambucanum*	URM 7559 ^T^	LS398489	–	LS398519	[47]
*F. pernambucanum*	URM 6801	LS398483	–	LS398513	[47]
*F. persicinum*	CBS 479.83 ^T^	MN170495	MN170361	MN170428	[45]
*F. persicinum*	CBS 131780	MN170496	MN170362	MN170429	[45]
*F. scirpi*	CBS 447.84 ^NT^	GQ505654	GQ505566	GQ505832	[46]
*F. scirpi*	CBS 448.84	GQ505592	GQ505504	GQ505770	[46]
*F. serpentinum*	CBS 119880 ^T^	MN170499	MN170365	MN170432	[45]
*F. sulawesiense*	InaCC F940 ^T^	LS479443	LS479422	LS479855	[49]
*F. sulawesiense*	Indo186	LS479449	LS479426	LS479864	[49]
*F. tanahbumbuense*	InaCC F965 ^T^	LS479448	LS479432	LS479863	[49]
*F. tanahbumbuense*	NRRL 34005	GQ505629	GQ505541	GQ505807	[46]
*F. toxicum*	CBS 406.86 ^T^	MN170508	MN170374	MN170441	[45]
*F. toxicum*	CBS 219.63	MN170507	MN170373	MN170440	[45]
*F. camptoceras*	CBS 193.65 ^ET^	MN170450	MN170316	MN170383	[45]
*F. neosemitectum*	CBS 189.60 ^T^	MN170489	MN170355	MN170422	[45]

Note: species obtained in this study are in bold. Superscript “T”, “ET”, and “NT” represents ex-type, epi-type, and neotype species, respectively. “–” represents the absence of sequence data in GenBank.

## Data Availability

The DNA sequence data obtained from this study have been deposited in GenBank under accession numbers; *tef-1* (OP020920, OP020921, OP020922, OP020923), *cam* (OP020924, OP020925, OP020926, OP020927), and *rpb2* (OP020928, OP020929, OP020930, OP020931).

## References

[1] Saediman H., Alwi L.O., Rianse I.S., Taridala S.A.A., Salahuddin S., Indarsyih Y., Astuti R.W. (2020). Comparative profitability of melon and watermelon production in South Konawe District of Southeast Sulawesi. WSEAS Trans. Bus. Econ..

[2] Assefa A.D., Hur O.S., Ro N.Y., Lee J.E., Hwang A.J., Kim B.S., Rhee J.H., Yi J.Y., Kim J.H., Lee H.S. (2020). Fruit morphology, citrulline, and arginine levels in diverse watermelon (*Citrullus lanatus*) germplasm collections. Plants.

[3] Kesh H., Kaushik P. (2021). Advances in melon (*Cucumis melo* L.) breeding: An update. Sci. Hortic..

[4] Nuangmek W., Aiduang W., Suwannarach N., Kumla J., Kiatsiriroat T., Lumyong S. (2019). First report of fruit rot on cantaloupe caused by *Fusarium equiseti* in Thailand. J. Gen. Plant Pathol..

[5] Manivannan A., Lee E.S., Han K., Lee H.E., Kim D.S. (2020). Versatile nutraceutical potentials of watermelon—A modest fruit loaded with pharmaceutically valuable phytochemicals. Molecules.

[6] Perkins-Veazie P., Davis A., Collins J.K. (2013). Watermelon: From dessert to functional food. Isr. J. Plant Sci..

[7] Lester G.E., Hodges D.M. (2008). Antioxidants associated with fruit senescence and human health: Novel orange-fleshed non-netted honey dew melon genotype comparisons following different seasonal productions and cold storage durations. Postharv. Biol. Technol..

[8] Parle M., Singh K. (2011). Musk melon is eat-must melon. Int. Res. J. Pharm..

[9] Maoto M.M., Beswa D., Jideani A.I.O. (2019). Watermelon as a potential fruit snack. Int. J. Food Prop..

[10] Vella F.M., Cautela D., Laratta B. (2019). Characterization of polyphenolic compounds in cantaloupe melon by-products. Foods.

[11] Food and Agriculture Organization of the United Nations. https://www.fao.org/faostat/en/#home.

[12] Keinath A.P. (2011). From native plants in Central Europe to cultivated crops worldwide: The emergence of *Didymella bryoniae* as a cucurbit pathogen. HortScience.

[13] Li P.F., Ren R.S., Yao X.F., Xu J.H., Babu B., Paret M.L., Yang X.P. (2015). Identification and characterization of the causal agent of gummy stem blight from muskmelon and watermelon in East China. J. Phytopathol..

[14] Nuangmek W., Aiduang W., Suwannarach N., Kumla J., Lumyong S. (2018). First report of gummy stem blight caused by *Stagonosporopsis cucurbitacearum* on cantaloupe in Thailand. Can. J. Plant Pathol..

[15] Babadoost M., Zitter T.A. (2009). Fruit rots of pumpkin: A serious threat to the pumpkin industry. Plant Dis..

[16] Ezrari S., Lahlali R., Radouane N., Tahiri A., Lazraq A. (2020). First report of *Fusarium equiseti* causing pre- and postharvest fruit rot on zucchini in Morocco. J. Plant Pathol..

[17] García-Estrada R.S., Márquez-Zequera I., Tovar-Pedraza J.M., Cruz-Lachica I. (2020). First report of cucumber fruit rot caused by *Fusarium incarnatum* in Mexico. Plant Dis..

[18] Rahman M.Z., Ahmad K., Siddiqui Y., Saad N., Hun T.G., Hata E.M., Rashed O., Hossain M.I. (2022). First report of *Fusarium equiseti*, causing fruit rot disease of watermelon in Malaysia. Plant Dis..

[19] Wonglom P., Sunpapao A. (2020). *Fusarium incarnatum* is associated with postharvest fruit rot of muskmelon (*Cucumis melo*). J. Phytopathol..

[20] Li Y.G., Zhang R., Meng L., Ali E., Ji P., Zhang Q.F., Cui G.W. (2019). Occurrence of fruit rot of cantaloupe caused by *Fusarium equiseti* in China. Plant Dis..

[21] Lima E.N., Oster A.H., Bordallo P.N., Araújo A.A.C., Silva D.E.M., Lima C.S. (2021). A novel lineage in the *Fusarium incarnatum-equiseti* species complex is one of the causal agents of fusarium rot on melon fruits in Northeast Brazil. Plant Pathol..

[22] Oyedeji E.O., Arogundade O., Tairu F.M., Elum C.G. (2022). Identification and characterization of fungi pathogen causing fruit rot disease of watermelon (*Citrullus lanatus*). Arch. Phytopathol. Plant Prot..

[23] Tuttle McGrath M., Naqvi S.A.M.H. (2004). Diseases of Cucurbits and their Management. Diseases of Fruits and Vegetables.

[24] Li Y., Ji P. (2015). First report of fruit rot of watermelon caused by *Fusarium equiseti* in Georgia in the United States. Plant Dis..

[25] Rivedal H.M., Stone A.G., Johnson K.B. (2018). First report of *Fusarium culmorum* causing fruit rot of winter squash (*Cucurbita maxima*) in Oregon. Plant Dis..

[26] Suwannarach N., Khuna S., Kumla J., Tanruean K., Lumyong S. (2019). First report of *Lasiodiplodia theobromae* causing fruit rot on melon (*Cucumis melo*) in Thailand. Plant Dis..

[27] Wilkinson K., Grant W.P., Green L.E., Hunter S., Jeger M.J., Lowe P., Medley G.F., Mills P., Phillipson J., Poppy G.M. (2011). Infectious diseases of animals and plants: An interdisciplinary approach. Philos. Trans. R. Soc. B.

[28] Suwannarach N., Khuna S., Kumla J., Cheewangkoon R., Suttiprapan P., Lumyong S. (2022). Morphology characterization, molecular identification, and pathogenicity of fungal pathogen causing kaffir lime leaf blight in northern Thailand. Plants.

[29] Choi Y.W., Hyde K.D., Ho W.H. (1999). Single spore isolation of fungi. Fungal Divers..

[30] Crous P.W., Lombard L., Sandoval-Denis M., Seifert K.A., Schroers H.-J., Chaverri P., Gené J., Guarro J., Hirooka Y., Bensch K. (2021). *Fusarium*: More than a node or a foot-shaped basal cell. Stud. Mycol..

[31] Wang M.M., Chen Q., Diao Y.Z., Duan W.J., Cai L. (2019). *Fusarium incarnatum-equiseti* complex from China. Persoonia.

[32] Wang M.M., Crous P.W., Sandoval-Denis M., Han S.L., Liu F., Liang J.M., Duan W.J., Cai L. (2022). *Fusarium* and allied genera from China: Species diversity and distribution. Persoonia.

[33] Kornerup A., Wanscher J.H. (1978). Methuen Handbook of Colour.

[34] O’Donnell K., Kistler H.C., Cigelnik E., Ploetz R.C. (1998). Multiple evolutionary origins of the fungus causing Panama disease of banana: Concordant evidence from nuclear and mitochondrial gene genealogies. Proc. Natl. Acad. Sci. USA.

[35] Carbone I., Kohn L.M. (1999). A method for designing primer sets for speciation studies in filamentous ascomycetes. Mycologia.

[36] O’Donnell K., Sutton D.A., Rinaldi M.G., Sarver B.A.J., Balajee S.A., Schroers H.-J., Summerbell R.C., Robert V.A.R.G., Crous P.W., Zhang N. (2010). Internet-accessible DNA sequence database for identifying fusaria from human and animal infections. J. Clin. Microbiol..

[37] Edgar R.C. (2004). MUSCLE: A multiple sequence alignment method with reduced time and space complexity. BMC Bioinform..

[38] Hall T. (2004). Bioedit Version 6.0.7. http://www.mbio.ncsu.edu/bioedit/bioedit.html.

[39] Felsenstein J. (1985). Confidence limits on phylogenies: An approach using the bootstrap. Evolution.

[40] Stamatakis A. (2006). RAxML-VI-HPC: Maximum likelihood-based phylogenetic analyses with thousands of taxa and mixed models. Bioinformatics.

[41] Miller M.A., Pfeiffer W., Schwartz T. Creating the cipres science gateway for inference of large phylogenetic trees. Proceedings of the 2010 Gateway Computing Environments Workshop (GCE).

[42] Ronquist F., Teslenko M., van der Mark P., Ayres D.L., Darling A., Höhna S., Larget B., Liu L., Suchard M.A., Huelsenbeck J.P. (2012). MrBayes 3.2: Efficient Bayesian phylogenetic inference and model choice across a large model space. Syst. Biol..

[43] Darriba D., Taboada G.L., Doallo R., Posada D. (2012). jModelTest 2: More models, new heuristics and parallel computing. Nat. Methods.

[44] Rambaut A. (2019). FigTree Tree Figure Drawing Tool Version 131.

[45] Xia J.W., Sandoval-Denis M., Crous P.W., Zhang X.G., Lombard L. (2019). Numbers to names—Restyling the *Fusarium incarnatum-equiseti* species complex. Persoonia.

[46] O’Donnell K., Sutton D.A., Rinaldi M.G., Gueidan C., Crous P.W., Geiser D.M. (2009). Novel multilocus sequence typing scheme reveals high genetic diversity of human pathogenic members of the *Fusarium incarnatum-F. equiseti* and *F. chlamydosporum* species complexes within the United States. J. Clin. Microbiol..

[47] Santos A.C.S., Trindade J.V.C., Lima C.S., Barbosa R.N., Costa A.F., Tiago P.V., Oliveira N.T. (2019). Morphology, phylogeny, and sexual stage of *Fusarium caatingaense* and *Fusarium pernambucanum*, new species of the *Fusarium incarnatum-equiseti* species complex associated with insects in Brazil. Mycologia.

[48] Lombard L., van Doorn R., Crous P.W. (2019). Neotypification of *Fusarium chlamydosporum*—A reappraisal of a clinically important species complex. Fungal Syst. Evol..

[49] Maryani N., Sandoval-Denis M., Lombard L., Crous P.W., Kema G.H.J. (2019). New endemic *Fusarium* species hitch-hiking with pathogenic *Fusarium* strains causing Panama disease in small-holder banana plots in Indonesia. Persoonia.

[50] De Oliveira M.J., Laranjeira D., Câmara M.P.S., Laranjeira F.F., Armengol J., Michereff S.J. (2014). Effects of wounding, humidity, temperature, and inoculum concentrations on the severity of corky dry rot caused by *Fusarium semitectum* in melon fruits. Acta Sci. Agron..

[51] Safari Z.S., Ding P., Nakasha J.J., Yuso S.F. (2020). Combining chitosan and vanillin to retain postharvest quality of tomato fruit during ambient temperature storage. Coatings.

[52] Bika R., Baysal-Gurel F. (2021). Identification of *Fusarium commune*, the causal agent of postharvest zinnia meltdown disease in Tennessee. HortTechnology.

[53] Yi R.H., Lian T., Su J.J., Chen J. (2022). First report of internal black rot on *Carica papaya* fruit caused by *Fusarium sulawesiense* in China. Plant Dis..

[54] Zhang X.P., Xia J.W., Liu J.K., Zhao D., Kong L.G., Zhu X.P. (2022). First report of *Fusarium pernambucanum* causing fruit rot of muskmelon in China. Plant Dis..

[55] Araújo M.B., Moreira G.M., Nascimento L.V., Nogueira G.A., Nascimento S.R.C., Pfenning L.H., Ambrósio M.M.Q. (2021). Fusarium rot of melon is caused by several *Fusarium* species. Plant Pathol..

[56] Lu M., Zhang Y., Li Q., Huang S., Tang L., Chen X., Guo T., Mo J., Ma L. (2022). First report of leaf blight caused by *Fusarium pernambucanum* and *Fusarium sulawesiense* on plum in Sichuan, China. Plant Dis..

[57] Laraba I., McCormick S.P., Vaughan M.M., Geiser D.M., O’Donnell K. (2021). Phylogenetic diversity, trichothecene potential, and pathogenicity within *Fusarium sambucinum* species complex. PLoS ONE.

[58] Pavlou G.C., Vakalounakis D.J., Ligoxigakis E.K. (2002). Control of root and stem rot of cucumber, caused by *Fusarium oxysporum* f. sp. *radicis-cucumerinum*, by grafting onto resistant rootstocks. Plant Dis..

[59] Shanmugam V., Veena K.H., Jain S., Tripathi M., Aggarwal R., Singh A.K. (2016). First report of seedling blight caused by *Fusarium solani* on cucumber from India. J. Plant Pathol..

[60] Gao X., Wang Y., Liu Y., Zhang M., Zhang W., Li Y. (2020). First report of leaf spot on cucumber caused by *Fusarium incarnatum* in China. Plant Dis..

[61] Leslie J.F., Summerell B.A., Ames I.A. (2006). The Fusarium Laboratory Manual.

[62] Rahjoo V., Zad J., Javan-Nikkhah M., Mirzadi Gohari A., Okhovvat S.M., Bihamta M.R., Razzaghian J., Klemsdal S.S. (2008). Morphological and molecular identification of *Fusarium* isolated from maize ears in Iran. J. Plant Pathol..

[63] Geiser D.M., Jiménez-Gasco M.M., Kang S., Makalowska I., Veeraraghavan N., Ward T.J., Zhang N., Kuldau G.A., O’Donnell K. (2004). FUSARIUM-ID v. 1.0: A DNA sequence database for identifying *Fusarium*. Eur. J. Plant Pathol..

[64] Nitschke E., Nihlgard M., Varrelmann M. (2009). Differentiation of eleven *Fusarium* spp. isolated from sugar beet, using restriction fragment analysis of a polymerase chain reaction-amplified translation elongation factor 1α gene fragment. Phytopathology.

[65] Jedidi I., Jurado M., Cruz A., Trabelsi M.M., Said S., González-Jaén M.T. (2021). Phylogenetic analysis and growth profiles of *Fusarium incarnatum-equiseti* species complex strains isolated from Tunisian cereals. Int. J. Food Microbiol..

[66] O’Donnell K., Ward T.J., Robert V.A.R.G., Crous P.W., Geiser D.M., Kang S. (2015). DNA sequence-based identification of *Fusarium*: Current status and future directions. Phytoparasitica.

[67] Balajee S.A., Borman A.M., Brandt M.E., Cano J., Cuenca-Estrella M., Dannaoui E., Guarro J., Haase G., Kibbler C.C., Meyer W. (2009). Sequence-based identification of *Aspergillus*, *Fusarium*, and *Mucorales* species in the clinical mycology laboratory: Where are we and where should we go from here?. J. Clin. Microbiol..

[68] Akram W., Ahmad A., Luo W., Yasin N.A., Wu T., Guo J., Wang Q., Li G. (2019). First report of stem and root rot of Chinese kale caused by *Fusarium incarnatum-equiseti* species complex in China. Plant Dis..

[69] Ismail S.I., Noor Asha N.A., Zulperi D. (2021). First report of *Fusarium incarnatum-equiseti* species complex causing leaf spot on rockmelon (*Cucumis melo*) in Malaysia. Plant Dis..

[70] Villani A., Moretti A., Saeger S.D., Han Z., Mavungu J.D.D., Soares C.M.G., Proctor R.H., Venâncio A., Lima N., Stea G. (2016). A polyphasic approach for characterization of a collection of cereal isolates of the *Fusarium incarnatum-equiseti* species complex. Int. J. Food Microbiol..

[71] Jeewon R., Hyde K.D. (2016). Establishing species boundaries and new taxa among fungi: Recommendations to resolve taxonomic am-biguities. Mycosphere.

[72] Li Y.G., Song X.L., Wang X.Q., Zhang H., Tian S., Ji P. (2018). First report of fruit rot of watermelon caused *by Fusarium equiseti* in China. Plant Dis..

[73] Kim J.W., Kim H.J. (2004). Fusarium fruit rot of posthavest oriental melon (*Cucumis melo* L. var. *makuwa* Mak.) caused by *Fusarium* spp. Res. Plant Dis..

[74] Ikediugwu F.E.O., Ogieva W.O. (1978). Fruit rot of *Citrullus lanatus* in Nigeria caused by *Fusarium solani*. Trans. Br. Mycol. Soc..

[75] Rampersad S.N. (2009). First report of *Fusarium solani* fruit rot of pumpkin (*Cucurbita pepo*) in Trinidad. Plant Dis..

[76] González V., Armengol J., Garcés-Claver A. (2018). First report of *Fusarium petroliphilum* causing fruit rot of butternut squash in Spain. Plant Dis..

[77] Li Y.G., Jiang W.Y., Jiang D., Wang R.T., Tian S., Ji P., Jiang B.W. (2019). First report of fruit rot on postharvest pumpkin caused by *Fusarium acuminatum* in China. Plant Dis..

[78] Hao F., Zang Q., Ding W., Ma E., Huang Y., Wang Y. (2021). First report of fruit rot of melon caused by *Fusarium asiaticum* in China. Plant Dis..

[79] Parra M.Á., Gómez J., Aguilar F.W., Martínez J.A. (2022). *Fusarium annulatum* causes Fusarium rot of cantaloupe melons in Spain. Phytopathol. Mediterr..

[80] Nuangmek W., Aiduang W., Kumla J., Lumyong S., Suwannarach N. (2021). Evaluation of a newly identified endophytic fungus, *Trichoderma phayaoense* for plant growth promotion and biological control of gummy stem blight and wilt of muskmelon. Front. Microbiol..

